# Langerin^+^ CD8α^+^ Dendritic Cells Drive Early CD8^+^ T Cell Activation and IL-12 Production During Systemic Bacterial Infection

**DOI:** 10.3389/fimmu.2018.00953

**Published:** 2018-05-07

**Authors:** Kelly A. Prendergast, Naomi J. Daniels, Troels R. Petersen, Ian F. Hermans, Joanna R. Kirman

**Affiliations:** ^1^Malaghan Institute of Medical Research, Wellington, New Zealand; ^2^School of Biological Sciences, Victoria University of Wellington, Wellington, New Zealand; ^3^Department of Microbiology and Immunology, University of Otago, Dunedin, New Zealand; ^4^Maurice Wilkins Centre for Molecular Biodiscovery, Auckland, New Zealand

**Keywords:** langerin, dendritic cell, diphtheria toxin, bacille Calmette–Guerin, systemic infection

## Abstract

Bloodstream infections induce considerable morbidity, high mortality, and represent a significant burden of cost in health care; however, our understanding of the immune response to bacteremia is incomplete. Langerin^+^ CD8α^+^ dendritic cells (DCs), residing in the marginal zone of the murine spleen, have the capacity to cross-prime CD8^+^ T cells and produce IL-12, both of which are important components of antimicrobial immunity. Accordingly, we hypothesized that this DC subset may be a key promoter of adaptive immune responses to blood-borne bacterial infections. Utilizing mice that express the diphtheria toxin receptor under control of the langerin promoter, we investigated the impact of depleting langerin^+^ CD8α^+^ DCs in a murine model of intravenous infection with *Mycobacterium bovis* bacille Calmette–Guerin (BCG). In the absence of langerin^+^ CD8α^+^ DCs, the immune response to blood-borne BCG infection was diminished: bacterial numbers in the spleen increased, serum IL-12p40 decreased, and delayed CD8^+^ T cell activation, proliferation, and IFN-γ production was evident. Our data revealed that langerin^+^ CD8α^+^ DCs play a pivotal role in initiating CD8^+^ T cell responses and IL-12 production in response to bacteremia and may influence the early control of systemic bacterial infections.

## Introduction

Bloodstream infections are commonly associated with significant morbidity and mortality, with substantial costs to health-care systems (1). Comprehensive knowledge of the essential constituents of an effective immune response to blood-borne bacterial exposure is, therefore, critical for reducing the associated burden to human health. The spleen is an important site for the induction of adaptive immunity. With extensive vasculature and associated lymphoid tissue, blood-borne bacteria or associated particulate components such as immune complexes are efficiently captured by a network of resident antigen-presenting cells (APCs) that include macrophages, B cells, and dendritic cells (DCs) (2). The acquisition of immunogenic elements and subsequent ligation of pathogen recognition receptors, primarily in the marginal zone of the spleen, drives activation of APCs, with some of these cells then moving to the white pulp to initiate adaptive immune responses. The precise roles of different APCs in promoting immune responses to infectious agents, however, have not been fully elucidated.

Splenic DCs can be broadly classified into conventional or plasmacytoid DCs, with conventional DCs further subdivided into three distinct populations: CD4^+^ DCs, CD8α^+^ DCs, and double negative DCs (3, 4). The CD8α^+^ DC population as a whole are reportedly efficient at cross-presenting antigen to CD8^+^ T cells (5, 6) and are major producers of IL-12, which is known to play a key role in promoting differentiation of IFN-γ-producing T cells (7, 8). However, despite their localization and ability to promote both IL-12 and CD8^+^ T cell responses, little is known about the importance of CD8α^+^ DCs in mediating immune responses to systemic bacterial infections.

Confounding the situation, however, it is recognized that there is heterogeneity in function between subpopulations of CD8α^+^ DCs. A subset of CD8α^+^ DCs expressing CX_3_CR1 has been identified, which lack the ability to produce IL-12 and cross-prime CD8^+^ T cells and have rearranged immunoglobulin genes—a feature more related to plasmacytoid DCs (9). The CD8α^+^ DC population can also be subdivided on the basis of expression of the c-type lectin receptor langerin (CD207). The langerin^+^ CD8α^+^ DCs in steady state are predominantly localized in the marginal zone (10, 11), a prime location for sampling bloodstream constituents. They have been shown to take up dying cells from the circulation and move to the T cell areas of the spleen to induce tolerance to acquired cell-associated antigens, a process thought to be involved in maintaining self-tolerance (11). However, in response to protein/adjuvant recognition, it was predominantly this langerin-expressing subset of CD8α^+^ DCs that produced IL-12 in the spleen, and these cells were critical for the priming of potent CD8^+^ T cell responses to circulating antigens (12). Given these characteristics, we hypothesized that the langerin^+^ CD8α^+^ DC subset may be involved in inducing protective responses to systemic infections. To address this hypothesis, we used knock-in mice expressing diphtheria toxin receptor (DTR) under the control of the langerin promoter (*lang*-DTREGFP mice) so that diphtheria toxin (DT) could be used to transiently deplete langerin-expressing DCs during blood-borne bacterial infection.

Bloodstream infections can be caused by a wide range of pathogenic microorganisms. *Escherichia coli, Staphylococcus aureus*, and *Streptococcus pneumoniae* are the most common causes of community-acquired bloodstream infection (13); however, mycobacterial species are also an important cause of bloodstream infection, particularly in immune-suppressed individuals (14–16). Since studies in mice depleted of CD11c^+^ DCs identified a crucial role for splenic DCs in mediating protective adaptive immunity after *Mycobacterium tuberculosis* (*Mtb*) infection (17), we chose to utilize a murine model of intravenous mycobacterial exposure.

To date, there is little information on which subgroups of DCs are important to the antimycobacterial response (18). However, as mice lacking either IFN-γ or IL-12p40 are highly susceptible to infection with *Mtb* (19–23), we considered it likely that the IL-12 producing capabilities of langerin^+^ CD8α^+^ DCs would contribute to control of a systemic mycobacterial infection. In addition, the ability of langerin^+^ CD8α^+^ DCs to cross-prime CD8^+^ T cells may be important in the context of mycobacterial infection as studies have shown that antigen-specific CD8^+^ T cells proliferate rapidly and contribute to immunity in the antimycobacterial response (21–24).

We report herein that during intravenous *Mycobacterium bovis* bacille Calmette–Guerin (BCG) infection, the depletion of langerin^+^ CD8α^+^ DCs led to a diminished immune response, with decreased serum IL-12p40 and delayed CD8^+^ T cell activation, proliferation, and IFN-γ production during infection. An increase in the bacterial burden in the spleen was also evident. These findings suggest that langerin^+^ CD8α^+^ DCs may play an important role in the response to blood-borne bacterial infection.

## Materials and Methods

### Mice

Male *lang*-DTREGFP (24), *lang*-EGFP (24), and C57BL/6J mice were bred and housed in the Biomedical Research Unit at the Malaghan Institute of Medical Research. Male *lang*-DTREGFP mice crossed with *lang*-EGFP mice (*lang*-DTREGFP × *lang*-EGFP) were used to better visualize GFP expression on langerin^+^ cells. OT-I and OT-II mice were crossed with B6.SJL-Ptprc^a^Pep3^b^/BoyJArc congenic mice to enable cell tracking through the congenic marker CD45.1. All mice were housed under specific pathogen-free conditions. All experiments were undertaken within the provisions of the Animal Welfare Act (1999) of New Zealand and approved by the Victoria University of Wellington Animal Ethics Committee.

### Mycobacteria

*Mycobacterium bovis* BCG Pasteur strain 1173P2 was grown at 37°C in Dubos broth (Difco, BD Diagnostics Systems, Sparks, MD, USA), supplemented with 10% Middlebrook oleic acid-albumin-dextrose-catalase (OADC) (Difco), until mid log phase and stored at −80°C in 0.05% PBS Tween80. For recombinant BCG-OVA (25) (a gift from Dr. James Triccas, University of Sydney, NSW, Australia), 50 µg/mL hygromycin (Roche, Manheim, Germany) was added. Before use, defrosted BCG stocks were sonicated briefly prior to dilution in PBS. BCG Pasteur and rBCG-OVA were injected intravenously (i.v.) in the lateral tail vein at 10^5^ CFU per mouse.

### Depletion of Langerin^+^ CD8α^+^ DCs In Vivo

*Lang*-DTREGFP mice were injected i.p. with 350 ng DT (Sigma-Aldrich, St. Louis, MO, USA) every 2 days for the period of time indicated in each experiment, commencing 2 days before BCG infection.

### Determination of Bacterial Burden

Spleens and livers were homogenized in PBS with 0.5% Tween80 (Sigma-Aldrich) and serial dilutions were plated on 7H11 agar (Difco) supplemented with 10% OADC, 25 mg carbenicillin and 100,000 U polymyxin B (Gibco, Invitrogen, Auckland, New Zealand). Plates were incubated at 37°C and bacterial counts performed after 2–3 weeks growth.

### Tissue Preparation

Spleens were digested in Liberase TL/DNAse I (Roche) in IMDM (Gibco, Life Technologies) for 30 min at 37°C. Cells were passed through a 70 µm strainer and red blood cells were lysed (Qiagen, MD, USA) before live cells were counted by Trypan blue exclusion.

### Flow Cytometry

Cells were blocked with anti-CD32/16 (clone 2.4G2, produced in-house) and stained with surface antibodies as indicated; CD8-PE (53-6.7), CD62L-APC (MEL-14), IFNγ-PE Cy7 (XMG1.2), CD11b APC CY7 (M1-70), and GR-1 FITC (RB6-8C5) from BD Pharmingen; B220-A647 (RA3-6B2), CD3-PE Cy7 (145-2C11), CD8-A700 (53-6.7), CD11c-eFluor450 (N418), CD44-PE Cy7 (IM7), CD45.1-PE (A20), Vα2-APC (B20.1) from eBioscience, CD11c-PE Cy7 (N418) from BioLegend. A viability dye, live/dead fixable blue (Invitrogen), was included before fixing cells with formalin (Sigma-Aldrich). Samples were collected on an LSRII SORP (BD, San Jose, CA, USA). Data were analyzed using FlowJo version 9.6 (Tree Star, Ashland, OR, USA).

### Adoptive Transfer of Carboxyflourescein Diacetate Succinimidyl Ester (CFSE)-Labeled OT-I and OT-II Cells

Spleens and lymph nodes from OT-I × B6 or OT-II × B6 mice were pooled and labeled with 2.5 µM CFSE (Molecular Probes, Eugene, OR, USA) at 37°C for 10 min. On day −1, 5 × 10^6^ cells were transferred intravenously to *lang*-DTREGFP recipient mice. On day 0, mice received 10^5^ CFU rBCG-OVA intravenously, and spleens were harvested at indicated time points to assess the CFSE proliferation profile by flow cytometry.

### *In Vitro* Re-Stimulation of OT-I Cells

Seven days after rBCG-OVA infection of OT-I transfer recipients, splenocytes were cultured with 1 µg/mL OVA_257–265_ (SIINFEKL) peptide (GenScript Corporation, Piscataway, NJ, USA) and 2 µg/mL anti-CD28 (clone 37.51, produced in-house) for 6 h at 37°C in complete IMDM (Gibco, Life Technologies), which contained 5% FCS (PAA Laboratories GmbH, Pasching, Austria), 1,000 µg/mL penicillin/streptomycin, 2 mM Glutamax, and 2-Mercaptoethanol (all Gibco, Invitrogen). 2 µM monensin (Sigma-Aldrich) was added for the last 4 h of incubation. Cells were fixed with formalin containing 4% formaldehyde (Sigma-Aldrich) and permeabilized with 0.1% Saponin buffer (Sigma-Aldrich) before being stained for intracellular IFN-γ, which was measured by flow cytometry.

### ELISA

Blood was collected at indicated time points from the lateral tail vein and left overnight to clot. The serum was separated by centrifugation and frozen at −20°C. IL-12p40 and IFN-γ ELISAs were performed following the manufacturer’s instructions (BD OptEIA) and the plate was read using a Versamax plate reader (Molecular Devices).

### Statistics

Bar graphs show mean + SEM error bars. For graphs displaying CFU (log_10_), the geometric mean + 95% CI is shown. Statistical significance was determined by one-way ANOVA with the Tukey posttest or Kruskal–Wallis test as indicated; significance within groups was determined by two-way ANOVA with the Bonferroni posttest. Graphpad Prism 5 software (Graphpad Software Inc., San Diego, CA, USA) was used for all analyses.

## Results

### Serum IL-12p40 Is Decreased, and Splenic Bacterial Burden Increased, in the Absence of Langerin^+^ CD8α^+^ DCs in BCG-Infected Mice

**Figure 1:**
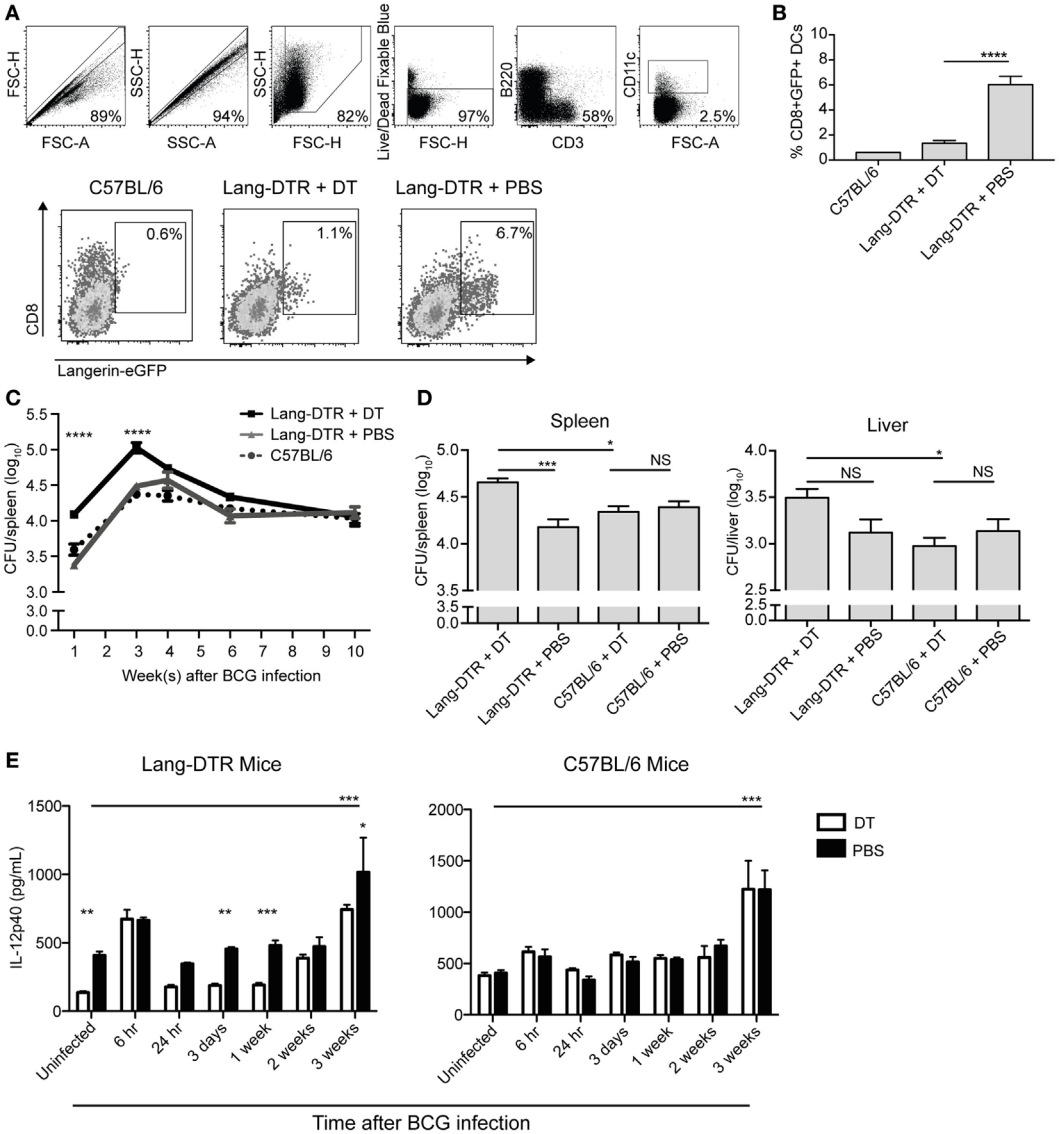
Serum IL-12p40 is decreased, and splenic bacterial burden increased, in the absence of langerin^+^ CD8α^+^ dendritic cells in bacille Calmette–Guerin (BCG)-infected mice. Mice were treated with 350 ng diphtheria toxin (DT) i.p. (or PBS as a control) every 2 days from day −2 to day 6 after BCG infection. **(A,B)** Day 7 after BCG infection, GFP expression in the spleens of lang-DTR × lang-EGFP mice and C57BL/6 controls was assessed by flow cytometry after gating on live CD3^−^ B220^−^ CD11c^+^ cells (*n* = 5 mice per group). ****p < 0.0001, one-way ANOVA. The results are representative of two pooled experiments. **(C,D)** On day 0, all groups of mice were infected with BCG i.v. At the times indicated, mice were culled and spleens and livers removed, homogenized, and plated on 7H11 agar. Colonies were counted after 2–3 weeks. **(C)** Spleens were then harvested from mice culled at 1, 3, 4, 6, and 10 weeks after BCG infection (*n* = 5–6 mice per group). ****p < 0.0001, two-way ANOVA between DT and PBS groups. **(D)** In an additional experiment, spleens and livers were harvested from mice culled 3 weeks after BCG infection (*n* = 10 mice per group). The results are representative of two independent experiments. *p < 0.05, ***p < 0.001, NS, not significant. **(E)** Mice were infected with BCG i.v. on day 0. At the times indicated, mice were tail-bled and serum IL-12p40 was measured by ELISA (*n* = 4–8 mice per group). *p < 0.05, **p < 0.01, ***p < 0.001, two-way ANOVA between DT and PBS-treated groups, ***p < 0.001, one-way ANOVA between uninfected and 3-week PBS-treated groups.

To determine if splenic langerin^+^ CD8α^+^ DCs were required for control of systemic BCG infection, we used *lang-*DTREGFP mice (referred to as Lang-DTR mice), which allowed depletion of langerin-expressing cells with DT during the course of infection. Multiple doses of DT were well tolerated and resulted in effective depletion of langerin^+^ CD8α^+^ DCs in the spleen [Figures 1A,B; (26)]. Depletion of langerin^−^ CD8α^+^ DCs was not evident (Figure S1 in Supplementary Material). Langerin^+^ CD8α^+^ DCs repopulated the spleen within 2–3 days of the final DT treatment (26).

To determine the effect of early depletion of splenic langerin^+^ CD8α^+^ DC on control of systemic blood-borne infection, the bacterial burden in the spleen was assessed over a 10-week period. BCG-infected, Lang-DTR mice were treated with DT from day −2 until day 6 of infection and spleens were harvested at 1, 3, 4, 6, and 10 weeks and then cultured to determine bacterial counts.

In langerin^+^ CD8α^+^ DC-depleted mice, a significant increase in spleen bacterial burden was evident 1 week after infection, compared to non-depleted mice, and this difference was maintained until 3 weeks after infection when spleen bacterial counts peaked (Figures 1C,D). In the later stage of infection, from 4 weeks onward, the difference in splenic bacterial burden between depleted and non-depleted mice was insignificant; by 10 weeks after infection, there was no discernible difference (Figure 1C). Extended depletion of langerin^+^ CD8α^+^ DCs (DT-treatment sustained until the 3-week peak of bacterial burden) had no further effect on the bacterial load in the spleen (Figure S2 in Supplementary Material).

Depletion of langerin^+^ CD8α^+^ DCs resulted in a small, but statistically insignificant, increase in the bacterial load of the liver compared to non-depleted mice (Figure 1D). DT treatment of C57BL/6 mice did not result in an increase in bacterial load in the liver or spleen (Figure 1D), confirming that the depletion of langerin^+^ CD8α^+^ DCs, rather than the DT treatment itself, was the cause of the increased bacterial burden during systemic BCG infection. These data may suggest the role of langerin^+^ CD8α^+^ DCs in the control of blood-borne bacterial infection is particularly important in, and potentially limited to, the spleen.

It has been reported in the CD11c-DTR mouse model that neutrophilia occurred after DT treatment in both naïve and bacterially infected mice, suggesting that neutrophilia was induced by DT itself (27). In the present study, an influx of neutrophils into the spleen was also observed early after BCG infection; however, this was significantly higher in DT-treated BCG-infected mice compared to DT-treated uninfected mice, suggesting that neutrophil influx was a response to BCG infection rather than DT treatment (Figure S3 in Supplementary Material). Indeed, uninfected mice treated with DT did not exhibit splenic neutrophilia.

IL-12p40 is essential for immune control of mycobacterial infections (19–21), and as such, the concentration in serum was measured during systemic BCG infection in the presence or absence of langerin^+^ CD8α^+^ DCs. Interestingly, the concentration of IL-12p40 was significantly lower in the serum of uninfected mice that were depleted of langerin^+^ CD8α^+^ DCs (Figure 1E), compared to non-depleted mice, suggesting that these DCs contribute to basal IL-12p40 production. After a transient increase in IL-12p40 in both DT-treated and non-depleted mice 6 h after infection, no significant increase in IL-12p40 above basal levels was measured until 3 weeks after infection, at which point the serum concentration was significantly higher in BCG-infected, non-depleted mice compared to uninfected controls; the depletion of langerin^+^ CD8α^+^ DCs resulted in reduced IL-12p40 production at this time point. Importantly, in C57BL/6 controls, DT treatment did not affect the serum concentration of IL-12p40, which was significantly increased 3 weeks after BCG infection.

To determine if the decreased IL-12p40 levels in infected langerin^+^ CD8α^+^ DC-depleted mice resulted in reduced IFN-γ production, serum IFN-γ was measured, although typically Th1 IFN-γ responses do not develop until 4 weeks after BCG infection (28). As anticipated, IFN-γ was undetectable in the serum early after BCG infection (weeks 1, 2, and 3; data not shown); however, at 4 weeks post BCG infection, when it was detected, the depletion of langerin^+^ CD8α^+^ DCs had no effect on IFN-γ levels in the serum (Figure S4 in Supplementary Material).

### Proliferation of OVA-Specific CD8^+^ T Cells in Response to Recombinant BCG-OVA Infection Is Delayed, and Activation Diminished, in the Absence of Langerin^+^ CD8α^+^ DCs

CD8α^+^ DCs are reportedly the most efficient splenic DC population for cross-presentation of antigen to CD8^+^ T cells (5, 6). It is also known that mice deficient in CD8^+^ T cells are impaired in their ability to control mycobacterial infections (29–31). Therefore, the proliferation of CD8^+^ T cells was assessed in the context of CD8α^+^ DC depletion. CFSE-labeled transgenic OVA-specific CD8^+^ T cells (OT-I cells) were adoptively transferred into Lang-DTR mice, followed by i.v. infection the next day with recombinant BCG that expressed the model antigen ovalbumin (rBCG-OVA). Control animals were infected with non-recombinant BCG, or were left uninfected (OT-I only controls). Animals were then treated with DT every 2 days for the first week of infection, or continuously until the end of the experiment (cont) as indicated, to deplete langerin-expressing cells.

**Figure 2:**
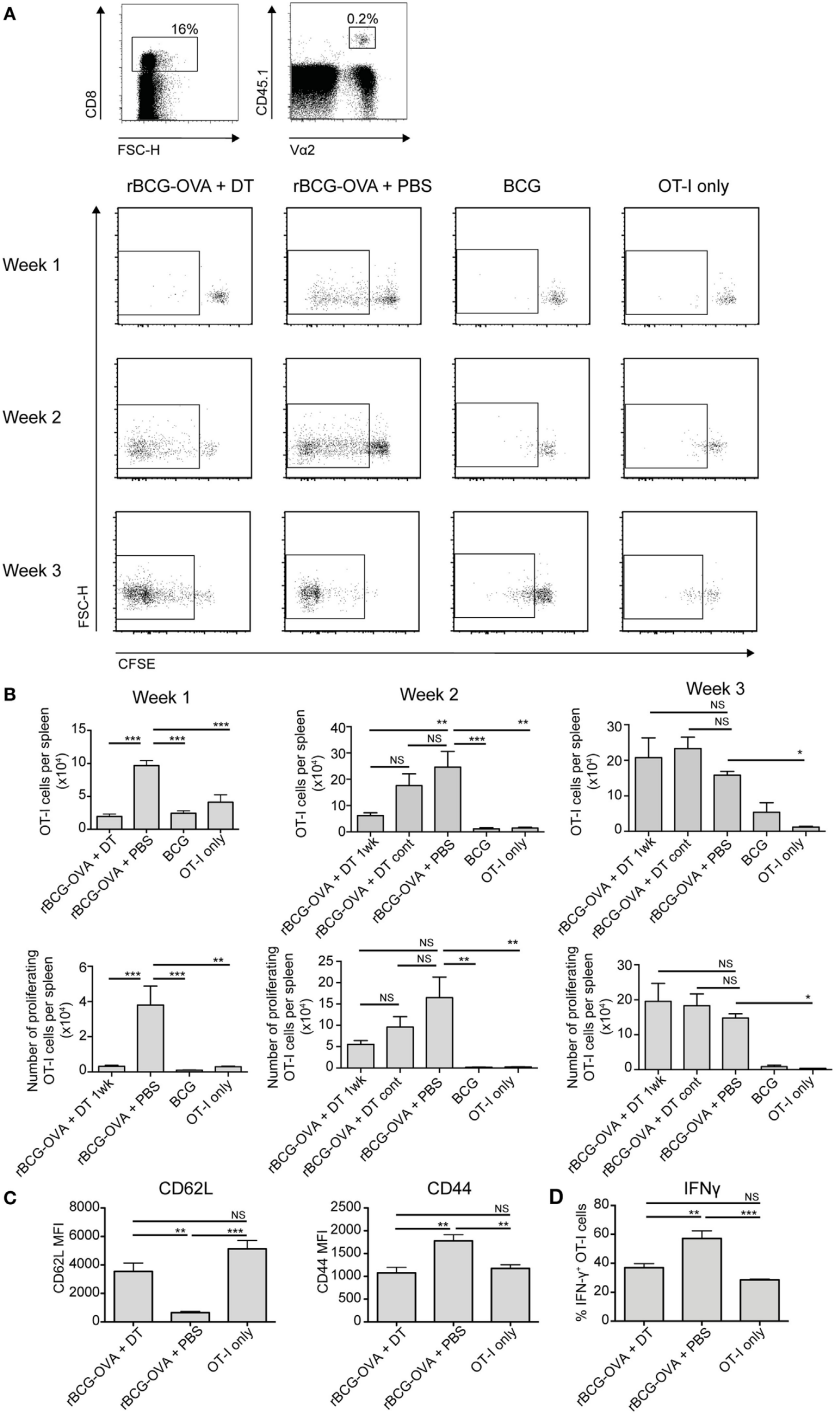
OT-I T cell proliferation in response to rBCG-OVA infection is delayed, and activation diminished, in the absence of langerin^+^ CD8α^+^ dendritic cells (DCs). Lang-diphtheria toxin receptor mice received 5 × 10^6^ carboxyflourescein diacetate succinimidyl ester (CFSE)-labeled splenocytes from OT-I × B6 donor mice 1 day before i.v. infection with rBCG-OVA or BCG. One group of mice were not infected (OT-I only group). Mice infected with rBCG-OVA were treated with 350 ng diphtheria toxin (DT) i.p. (or PBS as a control) starting on day −2, every 2 days for the first week of infection, or until the end of the experiment as denoted. At the indicated time points, mice were culled and spleens removed. The CFSE profile of splenic OT-I cells was assessed by flow cytometry. **(A)** Representative flow cytometry plots of the CFSE profile after gating on CD8^+^ CD45.1^+^ Vα2^+^ OT-I cells; flow plots show the proliferating cells in mice depleted with DT for 1 week. **(B)** The number of OT-I cells, and the number of proliferating OT-I cells within the spleen are shown, for mice receiving 1 week or continuous DT treatment (cont). **(C)** One week after rBCG-OVA infection, the median fluorescent intensity (MFI) of CD62L and CD44 expressed on OT-I cells was measured by flow cytometry. **(D)** One week after rBCG-OVA infection, splenocytes were cultured with 1 µg/mL OVA_257–265_ peptide for 6 h. Intracellular IFN-γ production by OT-I cells was measured by flow cytometry (*n* = 3–6 mice per group). *p < 0.05, **p < 0.01, ***p < 0.001, NS, not significant, one-way ANOVA. The results are representative of two independent experiments.

As expected (25), 1 week after rBCG-OVA infection, OT-I cells in the non-depleted recipient mice (rBCG-OVA + PBS) revealed diluted CFSE expression, indicative of proliferation (Figure 2A). In contrast, there was little proliferation at this time point in mice depleted of langerin^+^ CD8α^+^ DCs. Two weeks after infection, however, OT-I proliferation was diminished in DT-treated mice, and by 3 weeks after infection, OT-I proliferation occurred regardless of whether langerin^+^ CD8α^+^ DCs had been depleted (data not shown). Mice treated continuously with DT for the duration of the experiment did not have significantly different OT-I proliferation than mice depleted for just the first week of infection. These data suggest that depletion of langerin^+^ CD8α^+^ DCs delayed rather than prevented OT-I activation.

Infection with wild-type BCG did not induce significant OT-I proliferation compared to uninfected controls (OT-I only) at any time point assessed, confirming that the T cell activation was antigen-specific. The overall number of OT-I cells, and the percentage of proliferating cells, was significantly lower in the spleen of langerin^+^ CD8α^+^ DC-depleted mice 1 week after rBCG-OVA infection, as well as at 2 weeks after infection in terms of OT-I number, compared to non-depleted mice (Figure 2B). Three weeks after infection, however, OT-I cells in the spleen had increased significantly in response to rBCG-OVA infection in both depleted and non-depleted mice, compared to uninfected controls.

The apparently diminished CD8^+^ T cell response in the absence of langerin^+^ CD8α^+^ DCs was also reflected in the activation status and IFN-γ production of OT-I cells. One week after rBCG-OVA infection, OT-I cells in langerin^+^ CD8α^+^ DC-depleted mice displayed higher CD62L and lower CD44 compared to OT-I cells in non-depleted mice (Figure 2C), indicative of reduced activation. In addition, in OT-I cells isolated from mice depleted of langerin^+^ CD8α^+^ DC during infection, the proportion of IFN-γ^+^ OT-I cells in response to *in vitro* stimulation with OVA_257–265_ peptide was diminished compared to non-depleted mice (Figure 2D). Together, these data suggest that langerin^+^ CD8α^+^ DCs are important for early CD8^+^ T cell activation and function after BCG infection.

**Figure 3:**
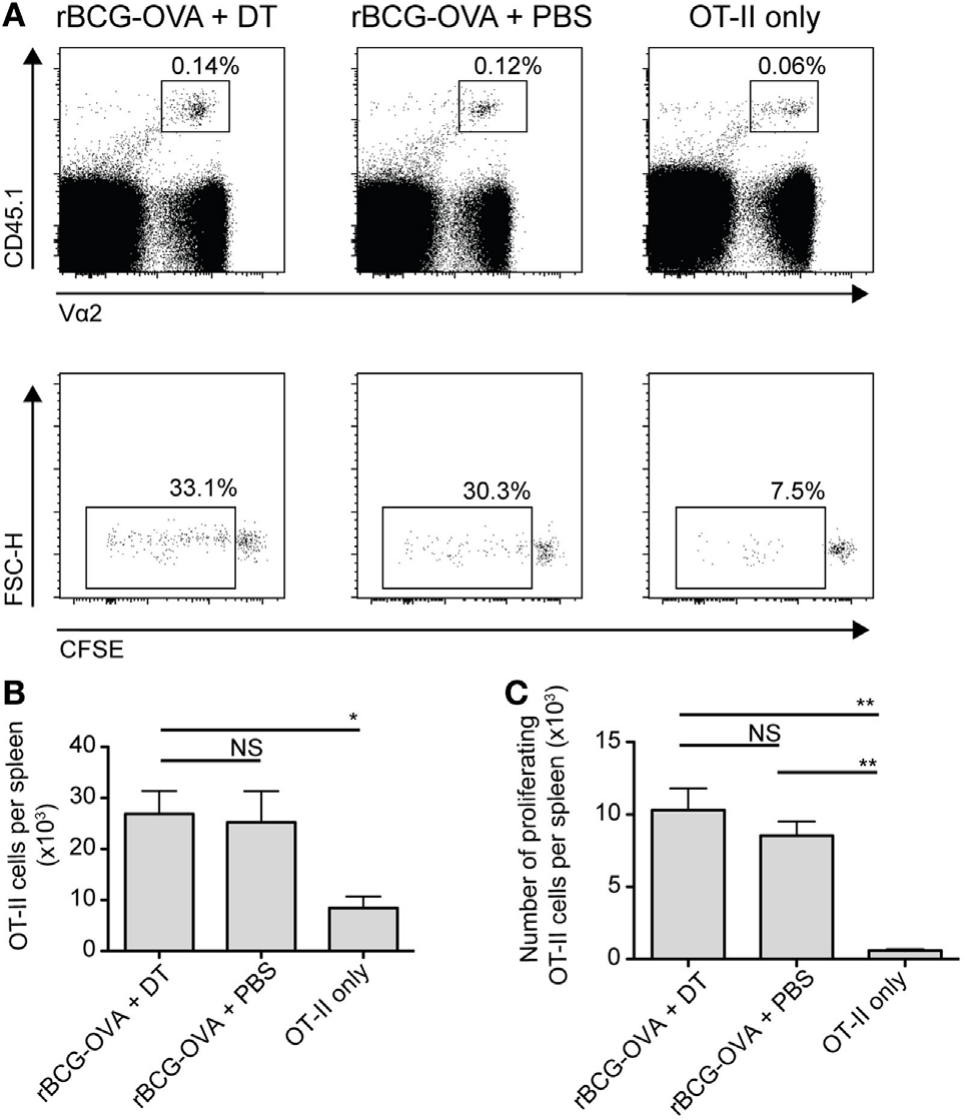
OT-II proliferation is unaffected by depletion of langerin^+^ CD8α^+^ dendritic cells. Lang-diphtheria toxin receptor mice received 5 × 10^6^ carboxyflourescein diacetate succinimidyl ester (CFSE)-labeled splenocytes from OT-II × B6 donor mice 1 day before i.v. infection with 10^5^ CFU rBCG-OVA. One group of mice was not infected (OT-II only group). Mice infected with rBCG-OVA were treated with 350 ng DT i.p. (or PBS as a control) starting on day −2, every 2 days until day 6. On day 9 after infection, mice were culled and spleens were removed. The CFSE profile of splenic OT-II cells was assessed by flow cytometry. **(A)** Representative plots of CD4^+^ CD45.1^+^ Vα2^+^ OT-II cells and their CFSE profiles, gated on proliferating (CFSE intermediate and negative) OT-II cells. **(B)** The number of OT-II cells and **(C)** the number of proliferating OT-II cells per spleen are shown (*n* = 3 mice per group). NS, not significant, *p < 0.05, **p < 0.01, one-way ANOVA. The results are representative of two independent experiments.

In analogous experiments, using adoptively transferred CFSE-labeled transgenic OVA-specific CD4^+^ T cells (OT-II cells), we did not observe any effect of langerin^+^ CD8α^+^ DC depletion on OT-II cell proliferation or the total number of OT-II cells in the spleen (Figure 3). As such, additional effects of depletion on these cells were not investigated further in this study.

## Discussion

Langerin^+^ CD8α^+^ DCs, resident in the marginal zone of the spleen, are localized for effective sampling of the blood; however, to the best of our knowledge, the role of these DCs in protection against systemic bacterial infection is yet to be reported. Here, we show that *in vivo* depletion of langerin^+^ CD8α^+^ DCs during intravenous BCG infection resulted in decreased IL-12p40 in the serum and a delay in antigen-specific CD8^+^ T cell proliferation, with reduced activation and IFN-γ production associated. An increased bacterial burden in the spleen was also evident. Together, these results suggest that langerin^+^ CD8α^+^ DCs may play an important role in the immune response against blood-borne bacterial infection.

In similar experimental contexts, several *in vivo* DC-depletion studies have demonstrated protective antimicrobial DC-mediated responses. Depletion of CD11c^+^ DCs during *S. aureus* bloodstream infection resulted in decreased serum IL-12, concomitant with an increased bacterial load (32). Similarly, bacterial numbers were increased after depletion of CD11c^+^ DCs during intravenous *Mtb* infection (17). Depletion of dermal langerin^+^ DCs in the context of *Leishmania major* infection resulted in a significant reduction in CD8^+^ T cell proliferation, with no effect on CD4^+^ T cell responses; however, no impact on parasite clearance was demonstrated (33).

By contrast, a number of reports have shown that depletion of DCs enhances, rather than impairs, host defense to the infective organism. A study of *Listeria monocytogenes* infection revealed a direct role for CD8α^+^ DCs in promoting bacterial disease. In the absence of CD8α^+^ DCs, bacteria were unable to traffic into the periarteriolar sheath and remained trapped in the marginal zone of the spleen, suggesting that the DCs facilitated entry of these microorganisms into the spleen. Not surprisingly, the absence of CD8α^+^ DCs during *L. monocytogenes* infection resulted in a reduced bacterial load compared to wild-type animals (34). Depletion of CD11c^+^ DCs prior to *Yersinia enterocolitica* infection also led to the surprising finding that animal survival increased; this was discovered to be due to neutrophil accumulation in the spleen following DC depletion, rather than a direct effect of the DCs (35). It appears, therefore, that while DCs are key players in the antibacterial response to blood-borne bacteria, specific DC subsets may be protective or detrimental to the immune response, depending on the infecting bacterial species.

The effect of depletion of CD8α^+^ DCs during systemic mycobacterial infection has not been reported, to date. In terms of the langerin^+^ CD8α^+^ DC subset, we have previously shown the functional specialization of these DCs *in vitro* (12), and *in vivo* (36) in protein/adjuvant models, particularly, with respect to enhanced IL-12 production. CD8α^+^ DCs, as a whole population, were superior to CD8α^−^ DCs in their ability to induce protective responses against BCG infection in an adoptive transfer setting (37), and have also been implicated as transient producers of IL-12p40 5 h after i.v. BCG exposure (38). In light of these findings, it could be expected that langerin^+^ CD8α^+^ DCs would be important for IL-12p40 production in the context of BCG infection; however, as our data show a transient increase of IL-12p40 6 h after infection in both DT-treated and non-depleted mice, it suggests that perhaps langerin^−^ CD8α^+^ DCs may be the early producers of IL-12p40, before langerin^+^ CD8α^+^ DCs are implicated by 3 weeks after infection.

The role of CD8α^+^ DCs has also been examined in *Batf3*^−/−^ mice. These mice lack the Batf3 transcription factor and, reportedly, as a consequence are deficient in CD8α^+^ DCs (39). However, as we and others have recently discovered, CD8α^+^ DCs do develop in these mice when bred on a C57BL/6 background under certain, as yet undefined, conditions (36, 40). We suggest that langerin^−^ CX3CR1^−^ CD8α^+^ cells that remain in *Batf3*^−/−^ mice are in fact precursors for the mature langerin^+^ population. Moreover, others have shown that infection with *Mtb, Toxoplasma gondii* or *L. monocytogenes* causes restoration of fully functional CD8α^+^ cDCs in *Batf3*^−/−^ mice, attributed to compensatory pathways involving the related transcription factors Batf and Batf2 (41). Therefore, the findings reported in this depletion study provide important novel insights into the role of langerin^+^ CD8α^+^ DCs in the immune response to blood-borne mycobacteria.

While we acknowledge that DT treatment also depletes Langerhans cells and langerin^+^ dermal DCs (24, 42), these cells do not have access to the spleen. Therefore, they likely play no role in the protection observed in non-depleted, BCG-infected mice. Furthermore, when comparing DT-treated Lang-DTR mice and splenectomized mice, CD8^+^ T cell activation in response to antigen and synthetic NKT cell ligand exposure is severely compromised in both models (43), indicating that the presence of the splenic langerin-expressing cells are crucial for promoting effective CD8^+^ T cell responses to circulating antigens. However, we cannot entirely discount the possibility that DT treatment led to a decrease in early killing efficiency by macrophages or DCs due to the uptake of apoptotic cells. It is also possible that the observed impact of depleting langerin-expressing cells in the context of BCG infection could have resulted from a reduction in a functional activity that can be attributed to the CD8α^+^ DC population as a whole. In this situation, it is possible the effect of depleting langerin-expressing cells could, therefore, simply reflect a large reduction [up to 60% (26)] of total CD8α^+^ DCs in spleens of these mice.

Interestingly, our work revealed that the depletion of langerin^+^ CD8α^+^ DCs lead to reduced CD8^+^ T cell activation and proliferation during the first week of BCG infection; however, 2 and 3 weeks after infection, CD8^+^ T cells proliferated in both depleted and non-depleted mice. As this effect was similarly apparent in mice treated with DT for either the first week or the full duration of the experiment, this suggested that langerin^+^ CD8α^+^ DCs primarily drive CD8^+^ T cell proliferation early after BCG infection, and may be redundant after this time. It is possible that other subsets of cross-presenting DCs, such as langerin^−^ XCR1^+^ DCs, were responsible for stimulating CD8^+^ T cells during these later time points, as these cells would not have been depleted in the langerin-DTR mice. In contrast, an increase in the spleen bacterial load was detected whether DT treatment was continued throughout the experiment or only administered for the first week after infection (in the latter case, DCs would have reconstituted during the second week). This suggests the role of langerin^+^ CD8α^+^ DCs in antimycobacterial control may be of particular significance in the first week after infection, and the importance of this early effect on the ensuing immune response is maintained throughout the bacterial proliferation phase.

Although significant insights into the beneficial role of langerin^+^ CD8α^+^ DCs in blood-borne bacterial infection are presented in this study, the mechanism of protection was not fully elucidated in this work. Interestingly, numerous reports have shown that *Mtb*-infected mice deficient in IL-12 or CD8^+^ T cells have increased bacterial burdens after 3–4 or 4–6 weeks of infection, respectively (20, 29, 31, 44). Therefore, in concordance with our findings, the IL-12 production and CD8^+^ T cell stimulation of langerin^+^ CD8α^+^ DCs may represent important aspects of the antibacterial response.

In this study, we report a previously unobserved role for langerin^+^ CD8α^+^ DCs during the initiation of the immune response against systemic mycobacterial infection. The functional heterogeneity of CD8α^+^ DCs has been underappreciated in the literature to date; thus, the data presented herein provide important insights specifically related to the langerin^+^ DC subset. These significant findings provide a platform for further investigations to more conclusively determine the mechanism of protective influence of langerin^+^ CD8α^+^ DCs, and whether this extends to other blood-borne bacterial infections.

## Ethics Statement

All experiments were undertaken within the provisions of the Animal Welfare Act (1999) of New Zealand and approved by the Victoria University of Wellington Animal Ethics Committee.

## Author Contributions

KP planned and performed all experimental work, analyzed and prepared figures, and assisted with writing of the manuscript; ND assisted with figure preparation and wrote the manuscript in consultation with KP and JK; TP assisted with experimental conception and analysis; IH assisted with experimental conception and planning; JK was in charge of overall direction and planning of the project, as well as experimental conception and manuscript preparation.

## Conflict of Interest Statement

The authors declare that the research was conducted in the absence of any commercial or financial relationships that could be construed as a potential conflict of interest.

## Supplementary Material

Figure S1 Diphtheria toxin (DT) treatment mediates specific depletion of langerin^+^ CD8α^+^ DCs. Mice were treated with 350 ng DT i.p. (or PBS as a control) every 2 days from day −2 to day 6 after bacille Calmette–Guerin (BCG) infection. On day 7 after BCG infection, GFP expression in the spleens of lang-diphtheria toxin receptor × lang-EGFP mice was assessed by flow cytometry after gating on live CD3^−^ B220^−^ CD11c^+^ cells (*n* = 5 mice per group), as per Figure 1A. **(A)** Gating strategy for langerin^+^ CD8α^+^ and langerin^-^ CD8α^+^ DC populations. **(B)** Bar graphs show the mean + SEM percentage of langerin^+^ CD8α^+^ and langerin^−^ CD8α^+^ DCs in mice treated with DT or PBS. NS, not significant, *****p* < 0.0001, one-way ANOVA. The results are representative of two pooled experiments.

Figure S2 Extended depletion of langerin^+^ CD8α^+^ DCs had no further effect on the bacterial load in the spleen. Mice were treated with 350 ng diphtheria toxin (DT) i.p. (or PBS as a control) every 2 days from day −2 to day 6 after bacille Calmette–Guerin (BCG) infection, or until 3 weeks after BCG infection. On day 0, all groups of mice were infected with BCG i.v. At 3 weeks after BCG infection, mice were culled and spleens removed, homogenized, and plated on 7H11 agar (*n* = 10–11 mice per group). Colonies were counted after 2–3 weeks. Graph shows the geometric mean of spleen bacterial CFU + 95% CI for mice treated with DT for 1 or 3 weeks, or PBS control. NS, not significant, ***p* < 0.01, one-way ANOVA. The results are representative of two independent experiments.

Figure S3 Diphtheria toxin (DT) treatment itself did not induce neutrophilia Lang-diphtheria toxin receptor mice were treated with 350 ng DT i.p. (or PBS as a control) on day −1. Bacille Calmette–Guerin (BCG) infection was carried out on day 0, and mice were culled and spleens removed 2 or 24 h after infection; uninfected mice were culled at the 24 h time point. **(A)** Representative flow cytometry plots of neutrophil proportions in BCG infected and uninfected mice, treated with DT or PBS. **(B)** The percentage of neutrophils (CD11b^+^ GR1^high^) in the spleens of BCG infected or uninfected mice, treated with DT or PBS is shown (*n* = 3–4 mice per group). NS, not significant, **p* < 0.05, ***p* < 0.01, ****p* < 0.001, one-way ANOVA.

Figure S4 Depletion of langerin^+^ CD8α^+^ DCs had no effect on IFN-γ levels in the serum 4 weeks after bacille Calmette–Guerin (BCG) infection Lang-diphtheria toxin receptor or C57BL/6 mice were infected with 10^5^ CFU BCG i.v. on day 0 and treated with 350 ng diphtheria toxin i.p. (or PBS as a control) starting on day −2 and continuing every 2 days for 1 week. Four weeks after BCG infection, mice were tail-bled and serum IFN-γ levels were measured by ELISA (*n* = 10 mice per group). NS, not significant, Kruskal–Wallis test. This result is representative of two independent experiments.
