# Supplementary figures and images for: Langerin+ CD8α+ Dendritic Cells Drive Early CD8+ T Cell Activation and IL-12 Production During Systemic Bacterial Infection
Source: Front Immunol. 2018 May 7;9:953. doi: 10.3389/fimmu.2018.00953 (PMC5949331; doi:10.3389/fimmu.2018.00953)

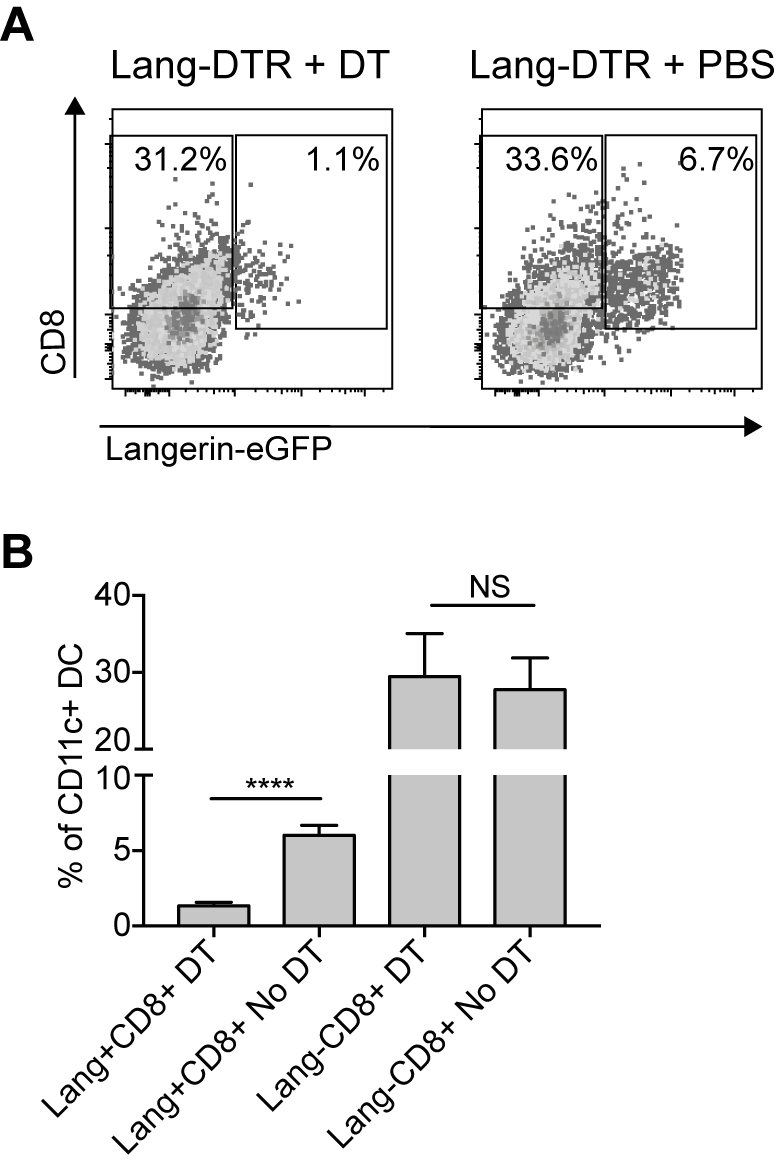

Supplement: Figure S1 — Diphtheria toxin (DT) treatment mediates specific depletion of langerin+ CD8α+ DCs. Mice were treated with 350 ng DT i.p. (or PBS as a control) every 2 days from day −2 to day 6 after bacille Calmette–Guerin (BCG) infection. On day 7 after BCG infection, GFP expression in the spleens of lang-diphtheria toxin receptor × lang-EGFP mice was assessed by flow cytometry after gating on live CD3− B220− CD11c+ cells (n = 5 mice per group), as per Figure 1A. (A) Gating strategy for langerin+ CD8α+ and langerin- CD8α+ DC populations. (B) Bar graphs show the mean + SEM percentage of langerin+ CD8α+ and langerin− CD8α+ DCs in mice treated with DT or PBS. NS, not significant, ****p < 0.0001, one-way ANOVA. The results are representative of two pooled experiments. [file image_1.TIF]

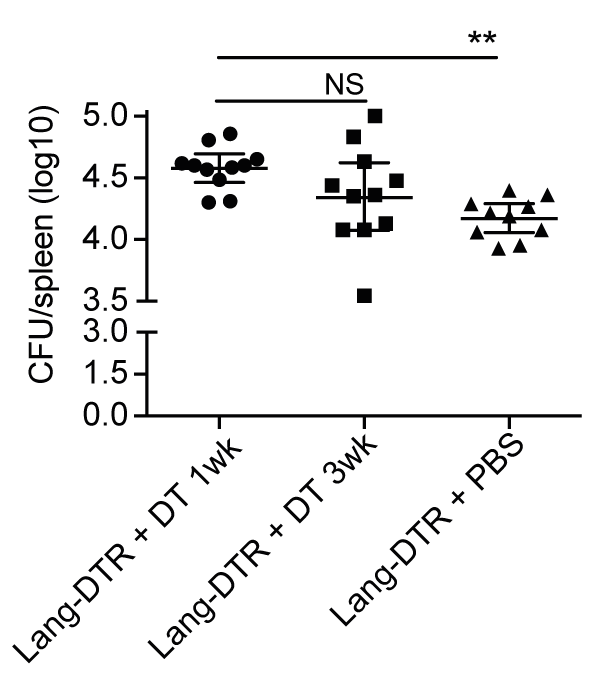

Supplement: Figure S2 — Extended depletion of langerin+ CD8α+ DCs had no further effect on the bacterial load in the spleen. Mice were treated with 350 ng diphtheria toxin (DT) i.p. (or PBS as a control) every 2 days from day −2 to day 6 after bacille Calmette–Guerin (BCG) infection, or until 3 weeks after BCG infection. On day 0, all groups of mice were infected with BCG i.v. At 3 weeks after BCG infection, mice were culled and spleens removed, homogenized, and plated on 7H11 agar (n = 10–11 mice per group). Colonies were counted after 2–3 weeks. Graph shows the geometric mean of spleen bacterial CFU + 95% CI for mice treated with DT for 1 or 3 weeks, or PBS control. NS, not significant, **p < 0.01, one-way ANOVA. The results are representative of two independent experiments. [file image_2.TIF]

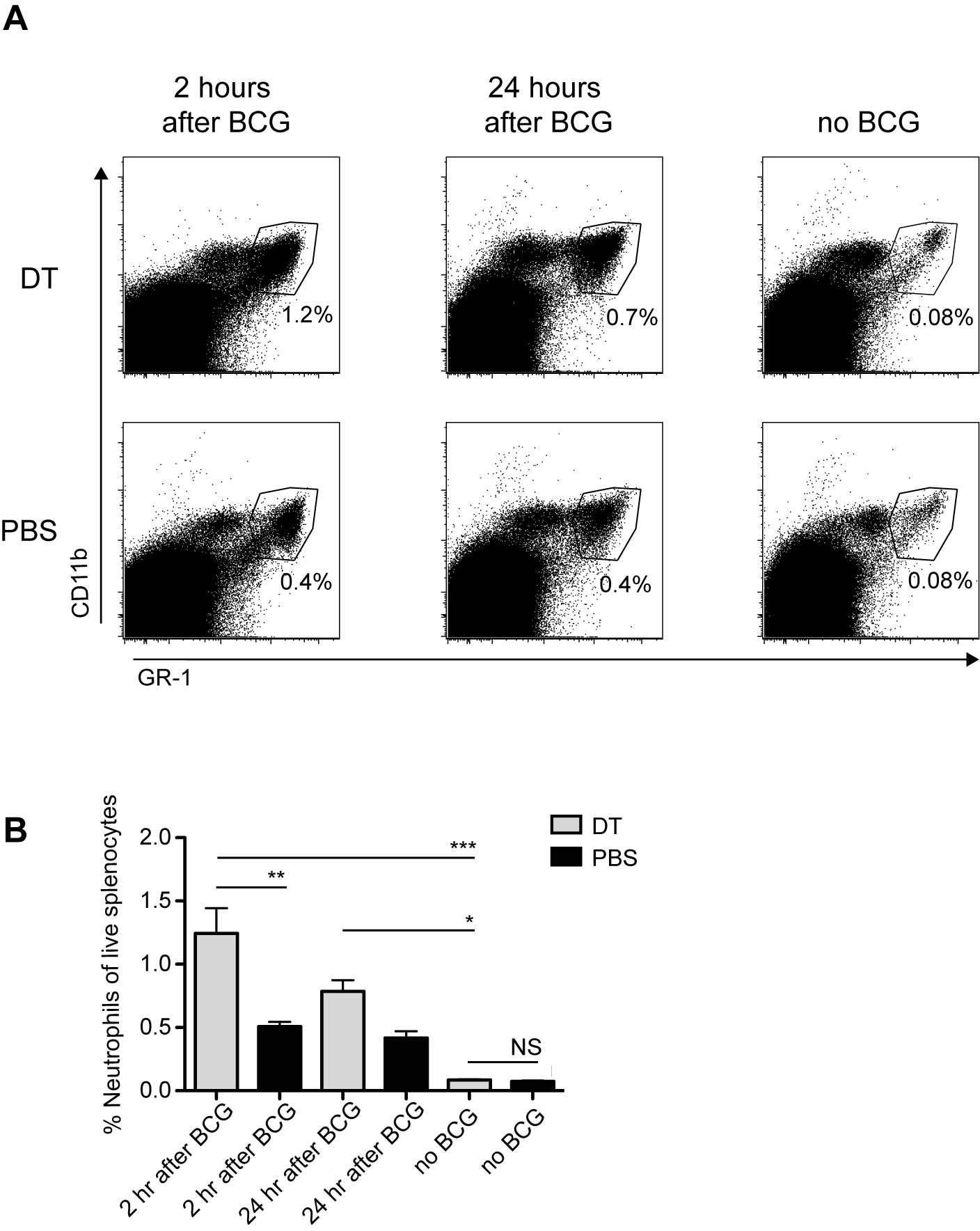

Supplement: Figure S3 — Diphtheria toxin (DT) treatment itself did not induce neutrophilia Lang-diphtheria toxin receptor mice were treated with 350 ng DT i.p. (or PBS as a control) on day −1. Bacille Calmette–Guerin (BCG) infection was carried out on day 0, and mice were culled and spleens removed 2 or 24 h after infection; uninfected mice were culled at the 24 h time point. (A) Representative flow cytometry plots of neutrophil proportions in BCG infected and uninfected mice, treated with DT or PBS. (B) The percentage of neutrophils (CD11b+ GR1high) in the spleens of BCG infected or uninfected mice, treated with DT or PBS is shown (n = 3–4 mice per group). NS, not significant, *p < 0.05, **p < 0.01, ***p < 0.001, one-way ANOVA. [file image_3.TIF]

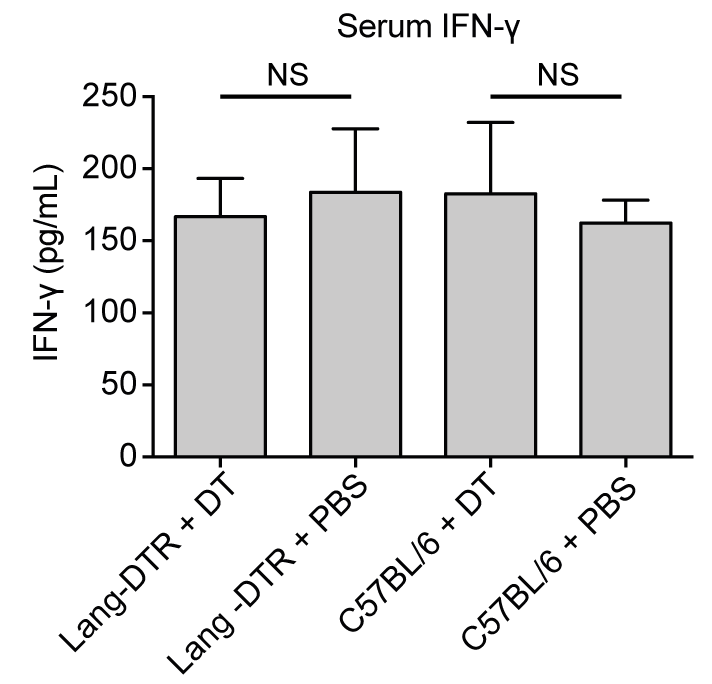

Supplement: Figure S4 — Depletion of langerin+ CD8α+ DCs had no effect on IFN-γ levels in the serum 4 weeks after bacille Calmette–Guerin (BCG) infection Lang-diphtheria toxin receptor or C57BL/6 mice were infected with 105 CFU BCG i.v. on day 0 and treated with 350 ng diphtheria toxin i.p. (or PBS as a control) starting on day −2 and continuing every 2 days for 1 week. Four weeks after BCG infection, mice were tail-bled and serum IFN-γ levels were measured by ELISA (n = 10 mice per group). NS, not significant, Kruskal–Wallis test. This result is representative of two independent experiments. [file image_4.TIF]
